# Regulation of the Immune System in Health and Disease by Members of the Bone Morphogenetic Protein Family

**DOI:** 10.3389/fimmu.2021.802346

**Published:** 2021-12-02

**Authors:** Tommaso Sconocchia, Giuseppe Sconocchia

**Affiliations:** ^1^ Division of Hematology, Medical University of Graz, Graz, Austria; ^2^ Institute of Translational Pharmacology, National Research Council (CNR), Rome, Italy

**Keywords:** BMP, cancer, inflammation, immune regulation, autoimmunity, dendritic cells, T cells, macrophages

## Abstract

Bone morphogenetic proteins (BMPs) are potent signaling molecules initially described as osteopromoting proteins. BMPs represent one of the members of the larger TGFβ family and today are recognized for their important role in numerous processes. Among the wide array of functions recently attributed to them, BMPs were also described to be involved in the regulation of components of the innate and adaptive immune response. This review focuses on the signaling pathway of BMPs and highlights the effects of BMP signaling on the differentiation, activation, and function of the main cell types of the immune system.

## Introduction

Bone morphogenetic proteins (BMPs) are a subfamily of signaling molecules that belong to the larger transforming growth factor-beta (TGFβ) family. The TGFβ family comprises more than 30 members which are structurally similar and share a similar signaling pathway. All the components of the TGFβ family signal *via* a system involving serine-threonine kinase receptors located on the cell surface. These receptors are divided into two types, type-I receptors, which are also called activin receptor-like kinases (ALKs), and type-II receptors. Seven type-I and five type-II receptors have been identified so far. TGFβ family members first bind to type-II receptors, which in turn phosphorylate the type-I receptors leading to the activation of intracellular SMAD proteins and consequently to gene transcription in the nucleus (1).

As their name implies, BMPs were first described for their ability to induce bone formation. Following this observation and thanks to advances in molecular biology, it was observed that BMPs do not only participate in bone formation but are endowed with numerous different functions (2). These include organ formation, tissue homeostasis, vascular remodeling, and regulation of immune responses (3, 4). The immunoregulatory function is one of the newest described functions of BMPs and is still not completely understood.

In this review, we discuss the current knowledge regarding the BMP signaling pathway and BMP-mediated immune regulation focusing on its effect on the various components of the immune system.

## BMP Signaling Pathway

BMPs are released by cells in an inactive precursor form. Once secreted, they are cleaved by protein convertases into their mature form that can bind to its receptor (5). To date numerous BMPs have been identified and can be classified into subgroups based on similarities in the coding region and amino acid sequence. The main subgroups include: BMP2/4, BMP5/6/7/8, BMP9/10, and BMP12/13/14 (also known as growth differentiation factor 5/6/7) (6).

**Figure 1:**
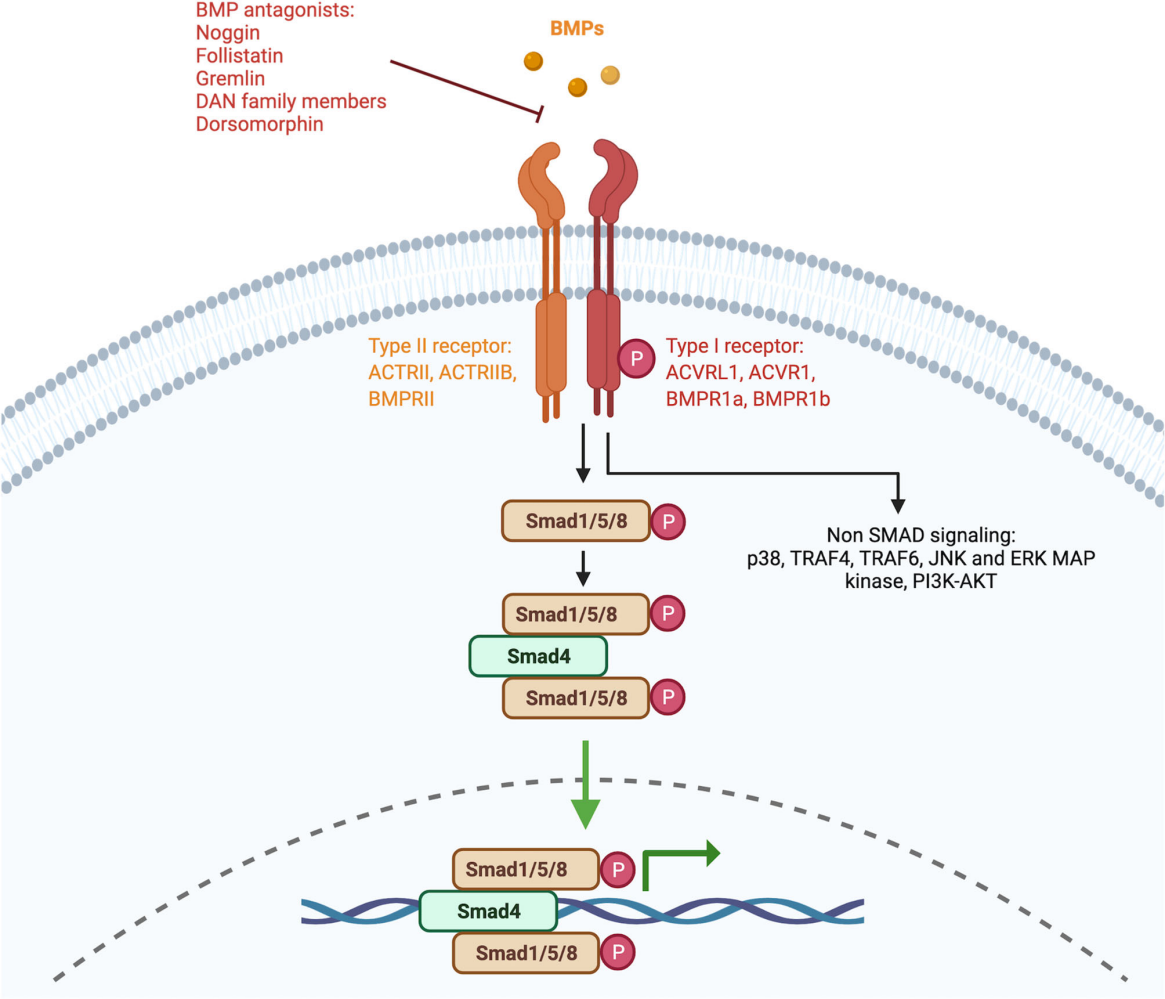
Signaling pathway of BMPs. Simplified schematic representation of the BMP signaling pathway. Members of the BMP family bind to serine/threonine receptors that are composed of type I and type II receptors which form heteromeric complexes. The signaling pathway can be divided into the canonical pathway and the non-canonical pathway. In the canonical pathway, also known as the SMAD-dependent pathway, SMAD1/5/8 proteins are phosphorylated and form complexes with a common SMAD4. In the non-canonical pathway, BMPs signal without the involvement of SMAD proteins. Created with BioRender.com.

The canonical BMP signaling pathway involves the participation of type-I and type-II receptors and SMAD complexes (7). The receptors can be shared between different members of the TGFβ family and the type-I receptors involved in BMP signaling include the activin receptor-like kinase 1 (ACVRL1)/ALK1, activin receptor type 1 (ACVR1)/ALK2, BMP receptor type 1a (BMPR1a)/ALK3, and BMPR1b/ALK6. The type-II receptors include BMP receptor type II (BMPR2), ACVR2A, and ACVR2B. BMPs act by forming heteromeric complexes by first binding to a type-II receptor which then recruits and activates the type I receptors. Following the formation of this complex, R-SMAD proteins (SMAD1, SMAD5, or SMAD8) are phosphorylated. The phosphorylated SMAD (pSMAD) proteins then bind to the common SMAD4 protein and this complex translocates to the nucleus where it promotes the expression of target genes (8) (**Figure 1**).

The affinity of the different BMP subgroups to the different type-I and type-II receptors varies. BMP2 and BMP4 were described to preferentially bind BMPR1a and BMPR1b. BMP6 and BMP7 were described to bind BMPR1a, BMPR1b (weakly), and ACVR1(strongly). BMP9 and BMP10 instead were described to bind to ACVRL1 and ACVR1. Lastly, the BMP12/13/14 group preferentially interacts with BMPR1b (1).

BMPs can also signal through a non-canonical pathway which involves the activation of type-I and type-II receptors but then proceeds independently from the SMAD proteins (9). This includes the involvement of components such as p38, TRAF4, TRAF6, JNK, and ERK MAP kinase, PI3K-AKT.

## BMP Regulation of the Immune System

**Figure 2:**
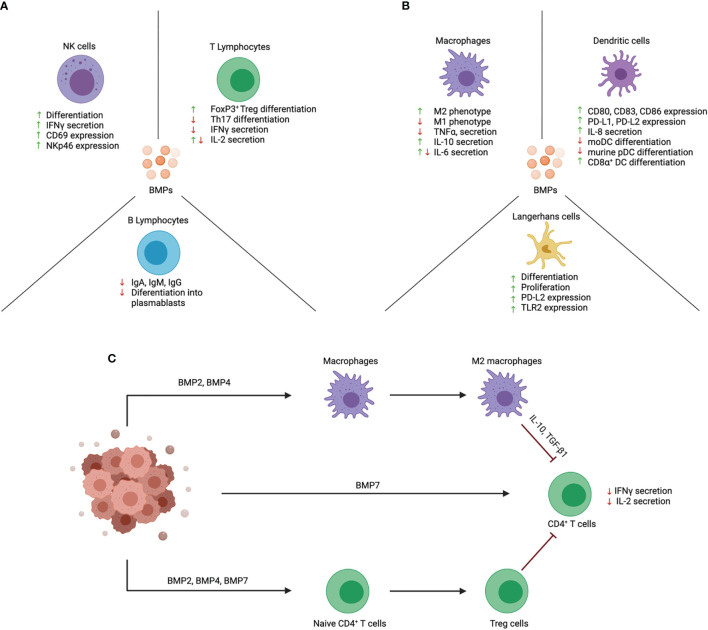
Effects of BMPs on function and differentiation of immune cell subsets. BMPs act upon a wide variety of immune cells that are of lymphoid and myeloid origin. **(A)** BMPs have an activating effect on NK cells and promote their cytotoxic function. Instead, BMPs exert an anti-inflammatory effect on T lymphocytes. BMPs inhibit the differentiation of naïve CD4^+^ T cells into Th17 cells and promote Treg cell differentiation. B lymphocyte function is also repressed by inhibiting their differentiation into plasmablasts and Ig production. **(B)** The effect of BMPs on myeloid cells is mainly an anti-inflammatory effect. BMPs skew macrophages towards the anti-inflammatory M2 phenotype. In DCs, BMPs are involved in their activation and promote the expression of co-inhibitory markers (PD-L1 and PD-L2). In addition, BMPs promote LC proliferation and differentiation. **(C)** Proposed scheme on how BMPs contribute to immune evasion in cancer. BMPs are secreted in the tumor microenvironment and may promote immune evasion by inhibiting the function of tumor-infiltrating immune cells. BMPs can act directly on T cells and inhibit their function. BMPs also act by promoting immunosuppressive cells like Treg cells and M2 macrophages which in turn can inhibit effector T cell function. Created with BioRender.com.

Various components of the immune system express BMP ligands, and their receptors (4). Although the effects of BMP signaling on the immune system are less studied in comparison to TGFβ, numerous new studies have described interesting and sometimes also conflicting immunoregulatory effects of BMPs. The main effects of BMPs on lymphoid cells are illustrated in **Figure 2A** and the effects on myeloid cells in **Figure 2B**.

### Dendritic Cells (DCs)

DCs secrete BMP ligands and express type-I receptors (BMPR1a, ACVR1, and to a lesser extent BMPR1b), type-II receptors (BMPRII), and the BMP downstream signaling components SMAD1, SMAD5, SMAD8, and SMAD4 (10). Numerous evidence supports the notion that BMP signaling is involved in the process of DC maturation. Treatment of monocyte-derived (mo)DCs with BMP ligands promoted the expression of maturation markers CD80, CD83, and CD86 and promotes IL-8 secretion (10). Besides increasing the expression of co-stimulatory markers, it was also described to increase the expression of the co-inhibitory markers programmed death-ligand (PD-L1) and PD-L2 (11, 12). By inhibiting autocrine BMP signaling during moDC maturation with the small molecule BMP inhibitor dorsomorphin (DM) Martinez et al., could show that this resulted in lower expression of PD-L1 and PD-L2 without impacting the expression of other co-stimulatory markers in a process that is mediated through the IRF-1 transcription factor (11). Moreover, blockade of autocrine BMP signaling in mature moDCs resulted in stronger T cell and NK cell stimulatory ability (11). These observations could be significant for the improvement of DC-based immunotherapies.

On top of regulating DC maturation, BMPs have also been reported to influence the differentiation of different DC subsets. The addition of conditioned media from BMP4-overexpressing acute lymphoblastic leukemia (ALL) cells inhibited the differentiation of moDCs from monocytes. Furthermore, moDCs treated with BMP4 displayed increased immunosuppressive functionality by expressing higher levels of IL-10 and TGFβ (13). In mice, BMP9 was shown to promote CD8α^+^DC differentiation and inhibit plasmacytoid DC (pDC) development in a process that is mediated through ACVRL1 (14).

The most studied and documented effect of BMPs on DC differentiation is probably represented by the role of BMP7 in the context of Langerhans cell (LC) differentiation. Before this observation, TGF-β1 was believed to be the key cytokine promoting LC differentiation *in vitro* and *in vivo.* LCs can be differentiated *in vitro* by culturing CD34^+^ hematopoietic progenitor cells (HPCs) in the presence of GM-CSF, SCF, FLT3L, TNFα, and TGF-β1 (15), and BMP7 was shown to be able to replace TGF-β1 in this process (16). Similarly to TGF-β1, BMP7 can also promote LCs from CD1c^+^ DCs (17). The observation that LC precursors are already present in developing skin that is devoid of active TGF-β1 but expresses BMP7 further supports the recently described role of BMP7 in LC differentiation. In addition, BMP7-deficient mice exhibited lower frequencies of LCs in the epidermis and these cells appeared to possess lower amounts of dendritic processes compared to their wild-type counterparts (16). Mechanistic studies performed by inhibiting TGFβ type I receptor (TGFβR1) and overexpressing BMPR1a additionally described that LC differentiation is driven by BMPR1a activation and that TGF-β1 promotes LC differentiation by signaling through BMPR1a. Blockade of TGFβR1 during *in vitro* LC differentiation led to increased numbers of LCs, whereas the overexpression of BMPR1a resulted in higher percentages and numbers of LCs (16). Follow-up studies focused on characterizing better these LCs differentiated in the presence of BMP7 (BMP7-LCs). BMP7-LCs expressed the hallmark LC markers CD207, CD1a, and E-cadherin like their TGF-β1 counterpart (TGF-β1-LCs). However, a more detailed analysis revealed that BMP7-LCs are phenotypically and functionally different from TGF-β1-LCs. BMP7-LCs exhibited a CD207^+^CD1c^+^CD206^+^TLR2^+^ phenotype which resembles that of LCs in psoriatic skin lesions (18). Functionally, BMP7-LCs secrete higher amounts of pro-inflammatory and anti-inflammatory cytokines in response to bacterial stimuli and possess a greater allostimulatory capacity of CD4^+^ T cells (16). Additionally, BMP7-LCs are stronger in promoting regulatory T (Treg) cell differentiation and naïve CD4^+^ T cells cultured in the presence of BMP7-LCs secrete lower amounts of IL-22 in comparison to naïve CD4^+^ T cells cultured with TGF-β1-LCs (12).

The role of BMPR1a in murine LC differentiation *in vivo* has still not been elucidated. A study performed on LCs from the oral mucosa, described a two-step sequential role for BMP7 and TGF-β1 in the process of LC differentiation. BMP7, expressed in the lamina propria, promoted the recruitment of LC precursors into the mucosal epithelium where TGF-β1 could finalize their differentiation (19). An analysis performed by us in mice lacking BMPR1a in the CD11c compartment (BMPR1a^fl/fl^CD11c^cre^ mice) revealed no significant differences in LC counts in the epidermis between knockout and wild-type mice in steady-state and during inflammation (12). However, it must be taken into consideration that the CD11c-cre is a useful tool for studying dendritic cell function and homeostasis. To have a better understanding of how BMPR1a affects dendritic cell differentiation, one should also investigate the effect of BMPR1a when its expression is deleted in earlier hematopoietic progenitors.

### Macrophages

Early studies investigating the role of BMPs in macrophages were performed on the murine macrophage cell line RAW 264•7. It was demonstrated that macrophages express ACVR1, and BMPR1a, and all three BMP type-II receptors (BMPR2, ACVR2A, and ACVR2B) (20). Functionally, treatment of RAW 264•7 cells and peritoneal macrophages with BMP6 promoted the expression of iNOS and IL-6. However, this effect was only detected with BMP6 and the same effect was not achieved with BMP2, BMP4, and BMP7 (21).

BMPs were also described to influence macrophage polarization. In murine models of prediabetic cardiomyopathy and atherosclerosis, BMP7 was observed to skew macrophage differentiation into the anti-inflammatory M2 phenotype with higher detectable levels of IL-10 and lower levels of IL-6, and TNFα (22, 23). BMP4 was also described to skew macrophage towards an M2 phenotype. In this case, its effect was investigated in human monocyte-derived macrophages and the context of tumor immunology and immunoevasion. BMP4 was detected in both conditioned media from bladder cancer cell lines and from, as previously described in the DC section, ALL cell lines. The addition of BMP4 and BMP4-containing conditioned media inhibited the pro-inflammatory M1 phenotype and promoted the M2 phenotype characterized by high IL-10 and low TNFα expression (13, 24). Pharmacological inhibition of BMP signaling in the cell lines THP-1 and RAW 264•7 differentiated towards the M2 macrophages caused distinct changes in markers associated with M1 and M2 phenotypes. This suggests a possible involvement of autocrine BMP signaling in macrophage polarization however, the obtained results were not consistent and only the M2 marker MMP12 showed a consistent decrease in the different cell lines treated with the BMP inhibitor DMH1 during M2 differentiation (25). This underlines the complexity of the BMP signaling pathway and the complexity of the polarization process of macrophages. Nevertheless, interesting effects of BMP signaling in macrophages were described particularly in the context of tumors where macrophages were described to play a role in tumor progression. These observations could in the future be exploited to develop new therapeutic targets.

### T Lymphocytes

T cells represent probably the immune cells in which the effects of BMPs have been the most studied. Numerous studies have focused on the role of BMP signaling in T cell activation, homeostasis, and polarization. The first investigations reported a role for BMPs in thymus organogenesis and thymocyte development. Thymic stromal cells were reported to secrete BMP2 and BMP4, and the BMP receptors and intracellular signaling components are expressed by CD4^-^CD8^-^ double-negative (DN) thymocytes (26). Inhibition of BMP signaling in thymic epithelial cells by transgenic expression of the BMP antagonist Noggin led to a smaller thymus with a low number of thymocytes that did not descend but remained at the level of the hyoid bone. The few reported thymocytes nevertheless, were phenotypically the same as the thymocytes in healthy fully developed thymuses (27). In addition, loss of BMP4 signaling in pharyngeal epithelial cells resulted in defective thymus morphogenesis (28). This suggests that BMP signaling plays a central role in the development of thymic stroma which indirectly regulates thymocyte differentiation. BMP signaling can also regulate thymocyte differentiation by direct signaling. The addition of exogenous BMP4 to fetal thymocyte organ cultures has been shown to negatively regulate thymocyte differentiation by inhibiting their transition from DN to CD4^+^CD8^+^ double-positive (DP) (29). *In vivo* studies performed using conditional knock-out mice in which BMPR1a expression is selectively deleted from the hematopoietic compartment (BMPR1a^fl/fl^vav^cre^ mice) revealed that BMP signaling is required for thymocyte development and that the effects are dose-dependent. Physiological levels of BMPR1a signaling are necessary for the correct differentiation however, elevated levels of BMPR1a signaling, obtained through the addition of exogenous BMP4, produced an opposite effect characterized by inhibition of the DN to DP stage (30).

BMP signaling was also described to be involved in the activation and homeostasis of mature CD4^+^ T cells. Naïve CD4^+^CD45RA^+^ T cells express mRNA for BMPR1a, ACVR1a, and the BMP intracellular components SMAD1, SMAD5, SMAD8, and SMAD4 (31). Despite this, at a protein level naïve CD4^+^ T cells BMPR1a is poorly expressed. Following TCR activation, BMPR1a expression increases significantly alongside CD25 expression suggesting that CD4^+^ T cells can respond to BMPs following activation (12, 31). Moreover, BMP signaling influences T cell activation and function by acting on the expression of IFNγ, IL-2, and CD25. Inhibition of the BMP pathway *in vitro* with DM resulted in lower secretion of IL-2 and inhibition of CD25 expression (12, 31, 32). In contrast to these observations, a recent study showed that activated CD4^+^ T cells treated with BMP7 secrete lower amounts of IL-2 (33). This suggests that the role of BMP signaling in IL-2 signaling is not fully elucidated and different BMPs or the activation of the different receptors could have varying effects on IL-2 production and secretion. BMP4 and BMP7 instead were both described to reduce IFNγ expression in activated CD4^+^ T cells (33, 34).

Besides their involvement in T cell activation and homeostasis, BMP signaling is additionally involved in Treg and Th17 differentiation. *In vitro* studies using murine naïve CD4^+^ T cells and DM indicated that BMP signaling promotes Th17 cell differentiation (32). However, more recent studies performed with murine BMPR1a-deficient CD4^+^ T cells and by treating murine CD4^+^ T cells with exogenous BMPs revealed that BMP signaling inhibits TGF-β-mediated Th17 cell differentiation (35). Regarding Treg differentiation, it was shown that BMPs can promote Treg differentiation. In support of this, it was observed that the treatment of murine naïve CD4^+^ T cells with DM under Treg-promoting conditions inhibited CD4^+^CD25^+^FoxP3^+^ Treg differentiation (32). In addition, BMP2 and BMP4 were described to have a synergistic effect in combination with TGF-β1 in the induction of Tregs (36). Murine studies using conditional knock-out mice in which BMPR1a is deleted from the CD4^+^ T cell compartment (BMPR1a^fl/fl^CD4^cre^ mice) further confirmed the role of BMP signaling in Treg differentiation. These mice displayed lower Treg numbers and in addition, CD4^+^ T cells isolated from these knock-out mice did not efficiently convert into Tregs (37, 38). The involvement of BMPs in Treg differentiation was also observed in the human setting with BMP7 being described to promote Treg differentiation from human naïve CD4^+^ T cells (12). This overall suggests that BMPs can influence the Th17/Treg cell balance and could be useful in Th17-driven autoimmune disorders. In fact, it was already shown to have a role in limiting inflammation in the DSS-induced colitis model (35) and would be of interest in studying whether it can also have a protective effect in other inflammatory diseases.

### B Lymphocytes

Very few studies investigating the role of BMPs in B cells have been performed. To date, only studies reporting data from *in vitro* experiments are available. Both naïve and memory B cells express the BMP type-II receptor ACVR2B. However, resting naïve B cells do not express BMP type-I receptors. They gain expression of ACVR1 following stimulation with CD40L/IL21. BMP2, BMP4, BMP6, and BMP7 were all demonstrated to have an inhibiting effect on CD40L/IL21-induced immunoglobulin (Ig) production. BMPs inhibited the production of IgM, IgA, and IgG by both naïve and memory B cells and also inhibited the differentiation of B cells into CD27^+^CD38^+^ plasmablasts (39). The effect of BMP7 was mainly due to its ability to promote apoptosis in the cells suggesting differential effects between BMP2/BMP4/BMP6, and BMP7 (40).

### Innate Lymphoid Cells (ILCs)

ILCs is one of the newest described family of immune cells. BMP signaling has also been implicated in ILC function and differentiation. Group 2 ILCs have been described to express BMPR1a, and BMPR1b (41) and to secrete BMP2 and BMP7 (42). Not much is known about the role of BMPs secreted from ILCs however, ILC-derived BMPs were suggested to regulate adipogenesis (41).

NK cells have been recently classified in the ILC family and represent the most studied ILC subset in terms of BMP signaling with studies pointing towards a role for BMPs in NK cell differentiation. Early CD34^+^BMPR1a^+^ thymic precursors express typical transcription factors (NFIL3, ID3) and cell surface markers (IL-2Rβ, IL-15RA, CD161) which are associated with NK cells (43–45). Reconstitution of thymic lobes from fetal SCID mice with CD34^+^BMPR1a^+^ precursors gave rise to high amounts of functional NK cells (about 60% CD3^-^CD161^+^CD56^+^ cells), whereas their reconstitution with CD34^+^BMPR1a^-^ counterparts failed to reach 10% CD3^-^CD161^+^CD56^+^. Moreover, *in vitro* differentiation experiments with the BMP inhibitor DM clarified that during their differentiation process NK cells produce BMP4 and that autocrine BMP signaling is involved in their differentiation (46). In addition to NK cell differentiation, the BMP signaling pathway also contributes to NK cell function. Blood circulating mature NK cells secrete BMPs (BMP2 and BMP6), express type I and type II receptors, and the BMP downstream signaling components SMAD1, SMAD5, SMAD8. Autocrine BMP signaling in NK cells was described to be important for optimal NK cell function by promoting IFNγ secretion, activation markers (CD69 and NKp46), and their ability to activate autologous DCs (47).

## Concluding Remarks

In the last decade, an increasing amount of studies are describing the immunomodulatory properties of BMPs. From these studies, it has emerged that BMPs play a crucial role in the development, activation, and suppression of the immune system. BMPs have been detected at aberrant levels in inflamed tissues and several tumors however, only a handful of studies have so far investigated their role in autoimmune diseases and cancer. One big open question remaining is whether they can be exploited as therapeutic targets for the treatment of cancer.

Their role in cancer remains incompletely understood, with studies describing both tumor-promoting and suppressing effects. This reflects the complexity of the BMP signaling pathway and how its effect is cell-dependent. Even though this two-sided effect has been described when investigating tumor cells (48), BMPs released in the tumor microenvironment act mainly in a tumor-promoting manner by promoting anti-inflammatory M2 macrophages, and by inhibiting T cell activation and function (**Figure 2C**). Immunotherapies like checkpoint inhibitors and chimeric antigen receptor (CAR)-T cells have revolutionized the treatment of cancer but unfortunately, these strategies have sometimes given unexpected or disappointing results. Modulation of the BMP signaling pathway may help improve the outcome of these therapies. Indeed, recently BMP7 was described to act as a mechanism responsible for resistance to anti-PD1 therapy (33). CAR-T cell therapy has shown to be effective in treating B cell malignancies (49) but has faced some challenges in the case of solid tumors mostly due to the anti-inflammatory tumor microenvironment rich in M2-macrophages and Treg cells which is not ideal for effector immune cell functions. Some new strategies to improve CAR-T cell therapy have been proposed [i.e. FCγ chimeric receptor T cells (50–52), armored CAR-T cells (53)] and these new strategies could be coupled with the inhibition of BMP signaling to promote a more robust immune response although, one must also be aware of the risk of inducing a strong autoimmune response. A similar strategy was already described for TGFβ, where disruption of TGFβRII signaling in CAR-T cells resulted in an increased anti-tumor response (54). The same could be investigated in the case of BMP receptors.

Future studies that aim at better characterizing the mechanisms of action of BMPs and how components of the TGFβ family regulate each other will strongly contribute to the development and improvement of current immunotherapeutic strategies.

## Author Contributions

TS conceived and designed the manuscript. All authors contributed to the article and approved the submitted version.

## Funding

GS is supported by the Italian Association for Cancer Research (AIRC): Investigator Grant (IG) 2020-24440, the National Operational Program (PON), “TITAN”, Ministry of the University and Research (MUR)-EU, and Rome Foundation project “Diabetes Mellitus, Regenerative and Reparative Processes, and Improvement of Pancreatic Beta Cell Function”. TS was trained within the Ph.D. program Molecular Fundamentals of Inflammation (DK-MOLIN) of the Medical University of Graz.

## Conflict of Interest

The authors declare that the research was conducted in the absence of any commercial or financial relationships that could be construed as a potential conflict of interest.

## Publisher’s Note

All claims expressed in this article are solely those of the authors and do not necessarily represent those of their affiliated organizations, or those of the publisher, the editors and the reviewers. Any product that may be evaluated in this article, or claim that may be made by its manufacturer, is not guaranteed or endorsed by the publisher.
